# Supplementary material for: Gut–Joint Axis: Impact of Bifidobacterial Cell Wall Lipoproteins on Arthritis Development
Source: Nutrients. 2023 Nov 21;15(23):4861. doi: 10.3390/nu15234861 (PMC10708502; doi:10.3390/nu15234861)
Supplement: Supplementary file 1 [file nutrients-15-04861-s001.zip › nutrients-2662415-supplementary.pdf]

Intestine	Distal Ileum			Caecum			Colon		
Treatment	Water	0.5 Lpps	1.0 Lpps	Water	0.5 Lpps	1.0 Lpps	Water	0.5 Lpps	1.0 Lpps
E. coli	3.1 ± 0.6 [a] (5) [b]	3.8 ± 0.7 (6)	4.0 ± 1.1 (7)	4.7 ± 0.7 (5)	5.1 ± 0.3 (6)	5.6 ± 1.1 *	4.6 ± 1.1 (5)	4.8 ± 0.8 (6)	5.0 ± 0.9 (7)
B. fragilis	3.1 ± 0.7 (6)	4.0 ± 0.3 (3)	3.8 ± 0.6 (3)	5.9 ± 1.4 (7)	6.2 ± 0.8 (6)	6.0 ± 0.5 (6)	5.6 ± 1.3 (7)	5.7 ± 0.4 (6)	5.8 ± 0.5 (6)
L. murinus	6.6 ± 1.2	6.3 ± 0.8 (7)	5.6 ± 1.2	7.8 ± 1.4	7.9 ± 0.2	5.4 ± 0.4 *	7.6 ± 0.3	7.7 ± 0.6	7.7 ± 0.7
L. reuteri	6.4 ± 0.8	6.3 ± 0.7 (7)	6.1 ± 0.8 (7)	8.2 ± 0.4	8.2 ± 0.5	8.3 ± 0.4	7.5 ± 0.5	7.7 ± 0.5	7.7 ± 0.4
L. johnsonii	6.1 ± 0.9	6.1 ± 0.8	5.4 ± 1.3	7.9 ± 0.5	7.9 ± 0.5	8.0 ± 0.3 (7)	7.4 ± 0.3	7.4 ± 0.4	7.3 ± 0.5 (7)
Ruminococcus sp.	5.7 ± 0.4 (7)	5.6 ± 0.4 (4)	6.0 ± 0.8 (5)	7.1 ± 0.3 (6)	6.5 ± 0.3 (5)	7.0 ± 0.6 (5)	6.1 ± 0.8 (6)	6.1 ± 1.1 (6)	6.8 ± 0.9 (4)

Table S1. Enumeration of selected bacteria at the end of CIA survey. [a]: mean ± SD (log cfu/g intestinal content), [b]: number of mice harboring detectable bacteria (out of 8 per group). *: significant difference in bacterial counts (see Figure 1D). *Parabacteroides goldsteinii*, *Akkermansia mucinophila*, *Eubacterium plexicaudatum*, *Pseudoflavonifractor* sp., *Lactobacillus intestinalis* (ASF360) as well as *Clostridium* sp (ASF356) were undetectable in most of the samples. Primers for *Mucispirillum schaedlerii* did not allow for the detection of the specific sequence.

Intestine	Distal Ileum			Caecum			Colon		
**Treatment**	**Water**	**Curative**	**Preventive**	**Water**	**Curative**	**Preventive**	**Water**	**Curative**	**Preventive**
P. goldsteinii	ND	4 (1)	ND	6.3± 0.4 (2)	ND	ND	6.5± 0.5 (4)	6.2 (1)	5.7 ± 0.9 (3)
A. mucinophila	5.1 ± 0.8 [a] (5) [b]	ND	5.8 ± 0.05 (2)	7.0± 1.5 (5)	7.3± 0.1 (2)	6.7± 1.3 (5)	8.3± 1.7 (3)	6.6± 1.3 (3)	6.8± 1.8 (5)
E. coli	4.2 (1)	5.4 ± 0.1 (2)	ND	ND	5.8 (1)	ND	3.9 (1)	4.7± 1.8 (2)	ND
B. fragilis	3.6 ± 0.6 (2)	4.1 ± 1.0 (3)	4.1 ± 0.7 (5)	5.7 ± 0.7	5.8 ± 0.6	5.7 ± 0.9	6.6 ± 0.5	6.1 ± 0.4	6.0 ± 0.9 (7)
L. murinus	6.3 ± 0.8	6.7 ± 0.9	6.8 ± 0.6	7.1 ± 0.5	7.6 ± 0.6	7.5 ± 1.0	8.1 ± 0.7	7.8 ± 0.6	7.5 ± 1.3
L. reuteri	7.2 ± 0.9	7.8 ± 1.1	7.3 ± 1.3	8.8 ± 0.4	9.1 ± 0.3	8.9 ± 0.8	9.3 ± 0.6	9.3 ± 0.5	9.2 ± 0.7
L. johnsonii	6.8 ± 1.0	7.5 ± 1.1	7.5 ± 1.4	8.7 ± 0.4	9.0 ± 0.4	9.2 ± 0.8	9.3 ± 0.7	9.1 ± 0.6	9.3 ± 0.7 (7)
M. schaedlerii.	5.7 ± 0.8 (7)	5.3 ± 0.4 (6)	6.4 ± 0.5 (6)	8.8 ± 0.9	8.9 ± 1.0	8.9 ± 1.4	8.5 ± 0.6	7.5 ± 0.8	8.3 ± 1.0 (7)
E. plexicaudatum	5.2 (1)	ND	5.5 (1)	6.3 ± 0.8 (6)	6.8 ± 0.9 (2)	7.2 ± 0.8 (5)	8.1 ± 0.6 (5)	ND*	6.9 ± 1.3 (4)

Table S2. Enumeration of selected bacteria at the end of CAIA survey. [a]: mean ± SD (log cfu/g intestinal content), [b]: number of mice harboring detectable bacteria (out of 8 per group). *Ruminococcus* sp. was undetectable in most of the samples. *: Curative treatment induced a drop in colon *E. plexicaudatum* (p = 0.0325 as compared with the control group).
